# Sets of Covariant Residues Modulate the Activity and Thermal Stability of GH1 β-Glucosidases

**DOI:** 10.1371/journal.pone.0096627

**Published:** 2014-05-07

**Authors:** Fábio K. Tamaki, Larissa C. Textor, Igor Polikarpov, Sandro R. Marana

**Affiliations:** 1 Departamento de Bioquímica, Instituto de Química, Universidade de São Paulo, São Paulo, SP, Brazil; 2 Instituto de Física, IFSC-USP, São Carlos, SP, Brazil; Russian Academy of Sciences, Institute for Biological Instrumentation, Russian Federation

## Abstract

The statistical coupling analysis of 768 β-glucosidases from the GH1 family revealed 23 positions in which the amino acid frequencies are coupled. The roles of these covariant positions in terms of the properties of β-glucosidases were investigated by alanine-screening mutagenesis using the fall armyworm *Spodoptera frugiperda* β-glycosidase (Sfβgly) as a model. The effects of the mutations on the Sfβgly kinetic parameters (*k*_cat_/*K*_m_) for the hydrolysis of three different *p*-nitrophenyl β-glycosides and structural comparisons of several β-glucosidases showed that eleven covariant positions (54, 98, 143, 188, 195, 196, 203, 398, 451, 452 and 460 in Sfβgly numbering) form a layer surrounding the active site of the β-glucosidases, which modulates their catalytic activity and substrate specificity via direct contact with the active site residues. Moreover, the influence of the mutations on the transition temperature (*T*_m_) of Sfβgly indicated that nine of the coupled positions (49, 62, 143, 188, 223, 278, 309, 452 and 460 in Sfβgly numbering) are related to thermal stability. In addition to being preferentially occupied by prolines, structural comparisons indicated that these positions are concentrated at loop segments of the β-glucosidases. Therefore, due to these common biochemical and structural properties, these nine covariant positions, even without physical contacts among them, seem to jointly modulate the thermal stability of β-glucosidases.

## Introduction

In recent years, the search for new enzymes using improvements in sequencing technologies has resulted in a large collection of protein sequences. For example, the glycoside hydrolase family 1 (GH1) groups have more than 5,000 β-glucosidase sequences in the CAZY database, only 274 of which have been marked as characterized to date [1]. Structural data - 41 crystallographic structures are available - and biochemical characterization of GH1 β-glucosidases revealed these enzymes share the same fold, the (β/α)_8_ barrel, and that their catalytic activity depends on a pair of glutamate residues, which act as acid/base and nucleophile in a double substitution mechanism [1]. Moreover, their companions, R and Y residues, are involved in the modulation of the p*K*_a_ of the catalytic glutamates. These catalytic residues are highly conserved among β-glucosidases, except for the myrosinases [2], [3]. Additionally, a network of hydrogen bonds formed by Q, H, W and E residues, which are placed and conserved in the active site of the β-glucosidases, modulates their substrate glycone specificity [4]–[7]. Finally, a set of variable residues, for which only the structural placement is conversed, forms the aglycone binding region for different β-glucosidases [8]–[10].

In parallel with the increase in sequence data, new methods to characterize the correlation between functions and structures of proteins have been developed that use special approaches to globally analyze protein sequences, and have revealed groups of residues that are jointly involved in determining functional properties. One of these methods is statistical coupling analysis (SCA), which, through covariation analysis of large multiple sequence alignments, is capable of identifying sets of residues that are important for protein folding [11], allostery [12], enzymatic activity and thermal stability [13]. Moreover, it was recently demonstrated that sets of covariant residues, termed sectors, are important starting points for protein engineering [14].

Among the characterized GH1 enzymes, the β-glucosidase from the fall armyworm *Spodoptera frugiperda* (Sfβgly) has been extensively studied, including biochemical and site-directed mutagenesis of the active site residues involved in pH optimum modulation and substrate specificity [2], [4], [10], [15], [16]. Additionally, residues outside of the active site that affect Sfβgly enzymatic activity *via* indirect contacts have been identified [17]. These findings make Sfβgly an excellent model for analyzing the function of covariant residues of the GH1 family.

We applied SCA to an alignment containing 768 β-glucosidase sequences and identified 23 covariant positions. Using Sfβgly as an experimental model, alanine residues were introduced at 18 of these covariant positions, and these single mutants were characterized for their thermal stability (*T*_m_) and kinetic parameters (*k*_cat_/*K*_m_) for the hydrolysis of three different chromogenic substrates. Based on the results, a set of 11 covariant positions, which are related to the enzymatic activity and form a layer surrounding the active site of the β-glucosidases, was identified. In addition, a second set of 9 covariant positions related to enzyme thermal stability, consisting of amino acid residues mostly at the loop regions of this (β/α)_8_ barrel structure, was identified.

## Materials and Methods

### Identification of the covariant positions in β-glucosidases

SCA was performed as described previously [11], [18] using a multiple sequence alignment containing 768 non-redundant β-glucosidases from the GH1 family, which were retrieved from the PFAM server (http://pfam.sanger.ac.uk). Site conservation (Δ*G^stat^*) and positional statistical coupling (ΔΔ*G^stat^*) parameters were calculated as previously described [19] using our own programs written in C/C^++^ [18]. Perturbations were considered significant when differences were present in at least 25% of the sequences in the alignment. A set of residue positions containing correlated conservations was obtained by clustering the ΔΔ*G^stat^* matrix using the Matlab (MathWorks) software package.

### Site-directed mutagenesis

Site-directed mutants were constructed using the QuikChange site-directed mutagenesis kit (Stratagene, La Jolla, CA, USA) following the manufacturer's instructions. Wild-type Sfβgly cloned into the pAE vector [20] was used as a template for PCR reactions performed with mutagenic primer pairs, which are presented on the Table S1. The pAE vector coding for wild-type Sfβgly, previously available, contains a T7 promoter, an ampicillin resistance mark and the insert was cloned in the *Nde*I and *Xho*I sites. Mutation incorporation was verified by DNA sequencing.

### Expression and purification of recombinant and wild-type Sfβgly

NovaBlue (DE3) competent cells (EMD Millipore, Billerica, MA, USA) were transformed with pAE plasmids encoding wild-type or mutant Sfβgly, plated on LB-agar containing ampicillin (50 µg/mL) and grown at 37°C for 16 h. Single colonies were grown at 20°C in LB broth containing ampicillin (50 µg/mL) until they reached an attenuance of 0.500 at 600 nm. Next, 0.4 mM (final concentration) isopropyl β-D-1-thiogalactopyranoside (IPTG) was added for 16 h to induce recombinant protein expression, after which cells were harvested by centrifugation (4,000×*g*, 20 min, 4°C) and frozen at −80°C. The pelleted cells were resuspended in 100 mM sodium phosphate pH 7.4 containing 200 mM NaCl, 60 mM imidazole and 10% (v/v) glycerol and were lysed with 3 ultrasound pulses (15 s, output 10 using a Branson Sonifer 250 adapted with a microtip) with 1 min intervals in ice to avoid heating the samples. The supernatants were recovered by centrifugation (13,200 g, 20 min, 4°C), and soluble recombinant proteins were incubated with 200 µL Ni-NTA Agarose (4°C, 1 h) (Qiagen, Hilden, Germany). The resin was pelleted and washed 5 times with 100 mM sodium citrate-sodium phosphate pH 6.0 containing 200 mM NaCl and 60 mM imidazole. Protein elution was performed using the same buffer containing 500 mM imidazole. Protein purity was verified by SDS-PAGE [21], and purified proteins were desalted using a minitrap G-25 (GE Healthcare, Upsala, Sweden) and stored at 4°C.

Protein concentration was determined by measuring the absorbance at 280 nm in 20 mM sodium phosphate pH 6.0 containing 6 M guanidinium hydrochloride. Extinction coefficients (ε_280__nm_) were calculated based on the primary sequences of the wild-type and mutant Sfβgly proteins using the ProtParam server at ExPaSy (http://web.expasy.org/protparam/). The extinction coefficients ranged from 2.149 to 2.053 (Table S2).

### Kinetic and thermal characterization of the mutant and wild-type Sfβgly

Enzyme kinetic parameters (*k*_cat_, *K*_m_ and k_cat_/*K*_m_) were determined for purified enzymes by measuring the initial rates (*v*_0_) of hydrolysis of at least 10 different concentrations of substrates, including *p*-nitrophenyl-β-D-glucopyranoside, *p*-nitrophenyl-β-D-fucopyranoside and *p*-nitrophenyl-β-D-galactopyranoside (Sigma, St. Louis, MO, USA) prepared in 100 mM sodium citrate – sodium phosphate buffer pH 6.0. Experiments were performed at 30°C. The hydrolysis of these substrates was detected following the formation of *p*-nitrophenolate by absorbance at 420 nm after addition of 250 mM sodium carbonate – sodium bicarbonate buffer pH 11 to the reaction samples. Kinetic parameters *K_m_* and *k_cat_* were determined by fitting the *v*_0_ and [S] data to the Michaelis-Menten equation using the Enzfitter software (Elsevier-Biosoft, Cambridge, UK).

Differential scanning fluorimetry (DSF) experiments of both wild-type and mutant Sfβgly were performed using SYPRO® Orange solution (500-fold dilution) (Sigma, St. Louis, MO, USA). Melting studies were performed in optical tubes using a 7500 Real-Time System (Applied Biosystems, Foster City, CA, USA). The temperature gradient ranged from 25°C to 95°C with a slope of 0.5% per step. The melting data were fitted according to recent literature [22], resulting in a theoretical *T_m_*. Fitting processes were performed using EnzFitter software.

### Tridimensional modeling and computational structure comparison

The tridimensional structure of Sfβgly was homology-modeled based on the crystallographic structure of the β-glucosidase from *Neotermes koshunensis* (PDB, 3VIK) using the Phyre2 software [23]. The sequences had 49% identity and 66% similarity. The structural models were visualized using the PyMOL molecular graphics system v1.1 (Schrödinger, LLC). The distances between amino acid pairs were calculated using DeepView/SwissPDBViewer v3.7 software [24]. The topology schemes of different β-glucosidases were manually drawn using information regarding the secondary structures visualized in PyMOL.

## Results and Discussion

The SCA [19] using 768 sequences of GH1 β-glucosidases revealed 23 covariant positions in their primary sequences (49, 54, 57, 62, 98, 112, 143, 176, 188, 195, 196, 203, 223, 278, 309, 329, 398, 445, 449, 451, 452, 456 and 460 in Sfβgly numbering). Except for position 451, involved in the β-glucosidase's specificity for the substrate glycone [4]–[7], none had been experimentally correlated with any biochemical or biophysical properties of the β-glucosidases.

Because SCA is based on the concept that covariant positions of a primary protein structure should be occupied by amino acid residues that are jointly involved in determining the same functional property [13], an experimental approach was designed to search the identified set of covariant positions for subgroups linked to the enzymatic activities or thermal stabilities of the GH1 β-glucosidases. Therefore, Sfβgly (GenBank code: AF 052729), a digestive enzyme from the fall armyworm *Spodoptera frugiperda* that has been extensively studied [4], [10], [15], was chosen as a representative β-glucosidase, and residues at its 23 covariant positions were separately replaced with alanine using a site-directed mutagenesis technique. Eighteen of these mutant enzymes were successfully expressed as recombinant proteins in NovaBlue (DE3) bacteria and purified (Figure S1). The E451A mutant of Sfβgly has been previously studied [4]. Mutant enzymes with replacements at positions 176, 329, 449 and 456 were not studied due to poor solubility.

The enzyme kinetic parameters for the hydrolysis of three different substrates catalyzed by wild-type and the 18 mutant Sfβgly proteins were determined (Table 1 and Figure S2). Additionally, the transition temperatures (*T*_m_) for the denaturation of the mutant and wild-type Sfβgly proteins were evaluated in Differential Scanning Fluorimetry (DSF) experiments (Table 2 and Figure S3).

**Table 1:** Enzyme kinetic parameters for the hydrolysis of *p*-nitrophenyl β-glycosides catalyzed by the wild-type and mutant Sfβgly proteins.

	NPβfuc			NPβglu			NPβgal		
Enzyme	*K*_m_ (mM)	*k*_cat_ (min^−1^)	*k*_cat_/*K*_m_ (min^−1^.mM^−1^)	*K*_m_ (mM)	*k*_cat_ (min^−1^)	*k*_cat_/*K*_m_ (min^−1^.mM^−1^)	*K*_m_ (mM)	*k*_cat_ (min^−1^)	*k*_cat_/*K*_m_ (min^−1^.mM^−1^)
**wild-type**	0.37±0.02	0.408±0.006	1.10±0.01	4.1±0.2	0.70±0.01	0.17±0.01	4.2±0.2	0.025±0.003	0.0060±0.0008
**K49A**	0.81±0.07	0.65±0.01	0.80±0.06	3.1±0.2	0.283±0.008	0.092±0.008	7.7±1	0.031±0.002	0.0040±0.0006
**W54A**	1.97±0.08	0.0530±0.0008	0.027±0.001	7.3±0.2	0.0279±0.0004	0.0038±0.0001	2.2 ±0.1	0.00073±0.00001	0.00033±0.00002
**M57A**	1.08±0.06	1.05±0.01	0.91±0.05	2.8±0.1	0.37±0.04	0.13±0.01	3.4±0.2	0.0266±0.0007	0.0079±0.0006
**P62A**	0.26±0.03	0.054±0.001	0.209±0.008	2.4±0.2	0.039±0.001	0.016±0.001	0.9±0.1	0.00195±0.00007	0.0021±0.0002
**F98A**	-	-	-	-	-	-	-	-	-
**N112A**	4.1±0.5	1.82±0.07	0.44±0.06	1.16±0.08	0.305±0.003	0.263±0.018	2.8±0.2	0.035±0.001	0.0125±0.0013
**W143A**	35±4	0.020±0.002	0.00057±0.00009	20±2	0.00124±0.00008	0.000062±0.000007	12±1	0.00026±0.00002	0.000022±0.000003
**P188A**	0.17±0.01	0.000239±0.000003	0.0014±0.0001	-	-	0.0000364±0.0000004	3.3±0.3	0.000237±0.000008	0.000073±0.000007
**G195L**	1.7±0.2	0.070±0.002	0.041±0.005	2.1±0.1	0.0404±0.0007	0.019±0.001	12±1	0.0113±0.0004	0.00094±0.00008
**Y196A**	2.7±0.2	0.205±0.006	0.076±0.006	10.2±0.6	0.115±0.003	0.0113±0.0007	13±1	0.0103±0.0004	0.00079±0.00006
**P203A**	0.87±0.08	0.215±0.007	0.25±0.02	50±5	0.52±0.04	0.010±0.001	-	-	0.00287±0.00001
**H223A**	0.90±0.06	0.23±0.01	0.26±0.02	5.7±0.5	0.025±0.00096	0.0044±0.0004	6.5±1	0.021±0.002	0.0032±0.0007
**P278A**	1.3±0.1	0.034±0.001	0.026±0.003	2.7±0.1	0.00250±0.00005	0.00093±0.00006	0.4±0.1	0.00058±0.00003	0.0145±0.0005
**P309A**	0.580±0.004	0.015±0.0002	0.026±0.001	1.9±0.1	0.0499±0.0009	0.026±0.002	4.4±0.2	0.0057±0.0001	0.00130±0.00008
**T398A**	-	-	-	-	-	-	-	-	-
**S445A**	1.30±0.09	0.39±0.01	0.30±0.02	1.58±0.07	0.317±0.003	0.200±0.009	5.0±0.3	0.46±0.01	0.092±0.007
**W452A**	-	-	-	-	-	-	-	-	-
**F460A**	9.1±0.5	0.046±0.001	0.0051±0.0003	58±11	0.013±0.001	0.00022±0.00005	11.6±0.7	0.00268±0.00008	0.00023±0.00001

- No activity.

**Table 2:** Transition temperatures (*T*_m_) for thermal denaturation of the wild-type and mutant Sfβgly proteins.

Enzyme	*T*_m_ (K)
wt	319
K49A	331.6
W54A	318.8
M57A	317.9
P62A	313.5
F98A	316.9
N112A	319.3
W143A	311.8
P188A	322.2
G195A	319
Y196A	319
P203A	319.8
H223A	325
P278A	324.7
P309A	311.9
S445A	317.1
W452A	316.2
F460A	306.8

Standard deviations were lower than 0.5 K.

Mutations resulting in at least a 4-fold change in the *k*_cat_/*K*_m_ for the hydrolysis of at least two different substrates were considered replacements of residues relevant for the enzymatic activity because such variation corresponds to a ΔΔ*G* ^‡^ higher than 3 *k*J/mol, which is equivalent to the disruption or formation of one hydrogen bond [25]. Based on these criteria, only 4 mutations had no significant effects on the enzymatic activity, namely K49A, M57A, N112A and S445A (Table 3).

**Table 3:** Mutational effects on the catalytic activity (*k*_cat_/*K*_m_ ratio) and thermal stability (Δ*T*_m_) of Sfβgly proteins.

Mutation position	*k_cat_*/*K*_m_ ratio (mutant/wt)			*ΔT_m_* (K)
	NPβglc	NPβgal	NPβfuc	
**49**	0.5	0.06	0.7	−7.2
**54**	0.02	0.005	0.02	−0.2
**57**	0.77	0.12	0.8	−1.0
**62**	0.09	0.03	0.18	−5.5
**98**	-	-	-	−2.1
**112**	1.5	0.19	0.38	−0.3
**143**	0.0004	0.0003	0.0005	−7.2
**188**	0.0002	0.0011	0.0012	3.2
**195**	0.11	0.01	0.03	0.1
**196**	0.06	0.01	0.06	0.0
**203**	0.06	0.04	0.21	0.8
**223**	0.30	0.05	0.075	6.0
**278**	0.005	0.02	0.02	5.8
**309**	0.15	0.02	0.02	−7.1
**398**	-	-	-	nd
**445**	1.1	1.5	0.27	−1.9
**452**	-	-	-	−2.8
**460**	0.0012	0.0037	0.0044	−12.2

Only mutational effects higher than a 4-fold change in the *k*_cat_/*K*_m_ ratio (0.25>*k*_cat_/*K*_m_ ratio >4) were considered significant for the enzymatic activity. Mutational effects on the thermal stability were considered relevant only for *ΔT_m_*>2.5 K. –, no activity; nd, not determined; wt, wild-type.

Similarly, the mutational effect on the thermal stability of Sfβgly was considered significant for *T*_m_ changes higher than 2.5 K, given that in the DSF experiments, this variation range corresponds to four-fold changes in the ratio of native to denatured wild-type Sfβgly. Based on that threshold, the mutations K49A, P62A, W143A, P188A, H223A, P278A, P309A, W452A and F460A were identified as replacements at positions involved in the thermal stability of Sfβgly (Table 3).

### SC positions related to the catalytic activity in β-glucosidases

**Figure 1:**
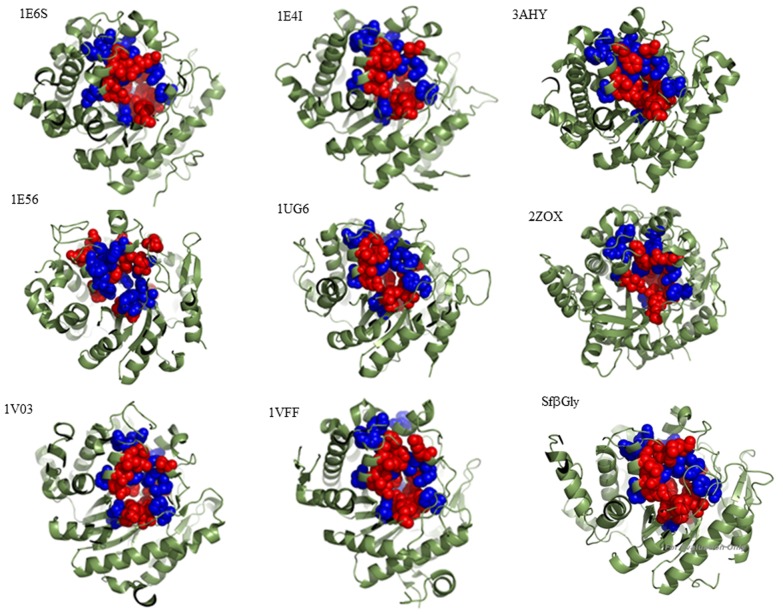
Structural comparison of β-glucosidases showing the active site residues (red) and sector A positions (blue). Myrosinase from *Sinapis alba* (1E6S); β-glucosidase A from *Paenibacillus polymyxa* (1EI4); β-glucosidase from *Trichoderma reesei* (3AHY); β-glucosidase Zmglu from *Zea mays* (1E56); β-glucosidase from *Thermus thermophilus* (1UG6); Human cytosolic β-glucosidase (2ZOX); SbDhr from *Sorghum bicolor* (1V03); β-glucosidase from *Pyrococcus horikoshii* (1VFF); β-glucosidase from *Spodoptera frugiperda* Sfβgly. The distances between sector A and the active site residues are shorter than 4.5 Å. The structures were visualized using PyMOL software.

Although no structural information was used in the SCA, visual inspection of the Sfβgly structural model revealed that residues at 11 positions related to the enzymatic activity are directly connected to active site residues through covalent or non-covalent bonds. Therefore, those 11 residues form a single group indirectly connected to each other through the active site residues and their non-covalent interactions with the substrate. This group was labeled sector A (Figure 1; Table 4). This observation is similar to previous SCAs of different protein families, which also showed groups of residues forming chains of interactions that connect distant points of their structures [19], [26]. In the particular case of serine proteases, two sets of covariant positions were identified. The first set contained residues involved in the catalytic mechanism (including the catalytic triad), and the second set contained residues present in the S1 pocket and was involved in substrate specificity [13].

**Table 4:** Residues from the active sites of β-glucosidases in direct contact with sector A positions.

Sector A position	Active site residues							
	SfβGly	1UG6	1E4I	1E56	1V03	1E6S	2ZOX	3AHY
**54**	K201^abr^, M453^abr^	H178^abr^, A394^abr^	H179, A407	F205^abr^, F466^abr^	L203^abr^, S462^abr^	D201, N466	F179^abr^, N426	F179, A426
**98**	R97^p^	R75	R77	R96	R97	R95	R75	R73
**143**	H142^bg^, E187*, E190^abr^, K201^abr^	H119^bg^, E164*, C167, H178^abr^	H121^bg^, E166*, C168, H179	H142^bg^, D191*, T194, F205^abr^	H143^bg^, D189*, T192, L203^abr^	H141^bg^, Q187, S190^abr^, D201	H120^bg^, Q165*, F179^abr^	H119^bg^, E165*, C168, F179
**188**	E187*, E190^abr^	E164*, C167	E166*, C168	D191*, T194	D189*, T192	Q187, S190^abr^	Q165*, V168^abr^	E165*, C168
**195**	E194^abr^	C167, L171^abr^, H178	C168, L172, H179	T194, F198^abr^, F205^abr^	T192, V196^abr^, L203^abr^	S190^abr^, R194^abr^, D201	M172^abr^, F179^abr^	C168, P172, F179
**196**	E194^abr^	L171^abr^	L172	F198^abr^	V196^abr^	R194^abr^	M172^abr^	P172
**203**	E194^abr^, K201^abr^	H178^abr^	H179	F205^abr^	L203^abr^	D201	F179^abr^	F179
**398**	R97^p^, E399*	R75, E338*	R77, E352*	R96, E406*	R97, E404*	R95, E409*	E373*	R73, E367*
**451**	Q39^bg^, W444^bg^, M453^abr^	Q18^bg^, W385^bg^, A394^abr^	Q20^bg^, W398^bg^, A407	Q38^bg^, W457^bg^, F466^abr^	Q39^bg^, W453, S462^abr^	Q39^bg^, W457^bg^, N466	Q17, W417^bg^, N426	Q16^bg^, W417^bg^, N426
**452**	Q39^bg^, H142^bg^, K201^abr^, M453^abr^	Q18^bg^, H119^bg^, H178^abr^, A394^abr^	Q20^bg^, H121^bg^, H180, A407	Q38^bg^, H142^bg^, F205^abr^, F466^abr^	Q39^bg^, H143^bg^, L203^abr^, S462^abr^	Q39^bg^, H141^bg^, N466	Q17, H120^bg^, F179^abr^, N426	Q16^bg^, H119^bg^, F179, N426
**460**	Y331^p^, W444^bg^	Y284, W385^bg^	Y296, W398^bg^	Y333, W457^bg^	Y331, W453^bg^	W457^bg^	Y309, W417^bg^	Y298, W417^bg^

The numbering of the sector A positions was based on the Sfβgly sequence. * identifies catalytic glutamic acids; ^p^ – indicates residues involved in the modulation of the p*K*_a_ of the catalytic glutamic acids; ^bg^ – shows residues involved in the binding of the substrate glycone; ^abr^ – indicates residues that form the aglycone binding region. Data regarding the role of individual residues in substrate binding and catalysis were retrieved from the literature [5], [27], [28], [29], [9], [30]. β-Glucosidase from *Spodoptera frugiperda* Sfβgly; β-glucosidase from *Thermus thermophilus* (1UG6); β-glucosidase A from *Paenibacillus polymyxa* (1E4I); β-glucosidase Zmglu from *Zea mays* (1E56); β-glucosidase SbDhr from *Sorghum bicolor* (1V03); myrosinase from *Sinapis alba* (1E6S); Human cytosolic β-glucosidase (2ZOX);β-glucosidase from *Trichoderma reesei* (3AHY).

Structural comparison showed that as in Sfβgly, the placement of sector A positions in close contact to the active site is also observed for different β-glucosidases from the GH1 family (Figure 1; Table 4). In brief, residues at sector A positions contact two glutamate residues (essential for catalysis), a pair of residues (tyrosine and arginine) involved in the modulation of the p*K*_a_ of the catalytic glutamate, and a group of residues that bind the substrates glycone and aglycone (Table 4). Thus, sector A residues might modulate several β-glucosidase properties, from catalytic activity to substrate specificity by affecting the positioning and properties of their active site residues. Moreover, data presented here suggest that the active site of the β-glucosidases is formed by a “layer” of highly conserved residues that interacts directly with the substrate and promotes its hydrolysis and is surrounded by a second “layer” formed by residues of the sector A positions (Table 4; Figure 1).

Evidence that joint variation of residues at sector A positions is involved in substrate binding and specificity is found when comparing β-glucosidases and 6-phospho-β-glucosidases, both groups belonging to the GH1 family. Indeed, 99% of the sequences exhibiting Y at position 460 also present S at position 451, and among these sequences, several were previously characterized as 6-phospho-β-glucosidases. Similarly, 99% of the sequences presenting E at position 451 present F at position 460, and several have β-glucosidase activity. Thus, the identities of residues at positions 451 and 460 are linked and directly connected to enzyme specificity. In 6-phospho-β-glucosidases, the replacement of E by S creates space in their active sites for the binding of substrates containing a phosphate group linked to the 6-OH of the glycone and also favors the formation of a hydrogen bond with this substrate. An additional hydrogen bond with the 6-phosphate group is formed by Y460 [31], [32] (Figure S4). Therefore, changing the substrate specificity of these two groups of β-glucosidases depends on replacements at both positions 451 and 460. A similar result was observed for the joint mutations C42A, C58A/V and S195T at trypsin covariant residues, which converted this enzyme from a serine to threonine protease [13].

Finally, five residues, P62, G195, Y196, P278 and P309, (Table 3) were not included in sector A because no direct or even indirect interactions connecting them to the active site were identified. Thus, the replacements of those residues affected the Sfβgly activity through currently unknown mechanisms.

### SC positions related to the thermal stability of β-glucosidases

**Figure 2:**
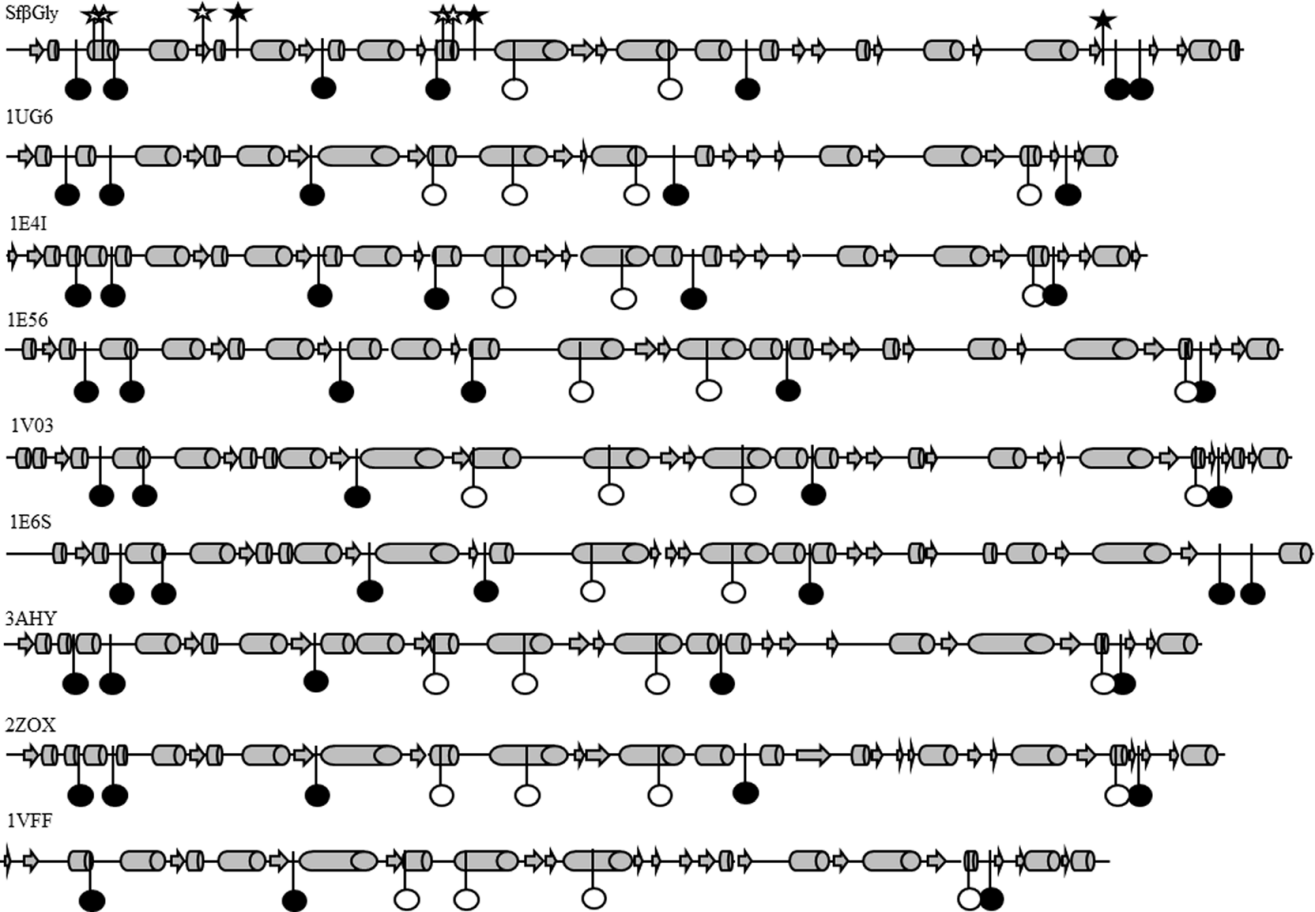
Distribution of sector S positions on the secondary structure of β-glucosidases. β-glucosidase from *Spodoptera frugiperda* Sfβgly; β-glucosidase from *Thermus thermophilus* (1UG6); β-glucosidase A from *Paenibacillus polymyxa* (1E4I); β-glucosidase Zmglu from *Zea mays* (1E56); β-glucosidase SbDhr from *Sorghum bicolor* (1V03); myrosinase from *Sinapis alba* (1E6S); β-glucosidase from *Trichoderma reesei* (3AHY); Human cytosolic β-glucosidase (2ZOX); β-glucosidase from *Pyrococcus horikoshii* (1VFF). α-Helices are represented by cylinders, β-strands by arrows and loops by lines. Sector S positions are shown as circles, whereas non-sector S positions are shown as stars. The symbols (circles or stars) in black indicate positions placed at loops, whereas white symbols mark positions at helices or strands.

The coupled positions in which replacements significantly affect Sfβgly *T*_m_ (Table 3) are mostly occupied by prolines (4 out 9 positions) or positively charged residues (K49 and H223). Conversely, only one proline residue was identified among the eight coupled positions not related to Sfβgly thermal stability, and no charged residue is present among them. In addition, sequence comparison shows that positions 62, 188, 278 and 309 (Sfβgly numbering) are predominantly occupied by proline residues among family 1 β-glucosidase sequences (frequencies higher than 50%), whereas positively charged residues are dominant at positions 49 and 223 (90% and 63%, respectively). Moreover, structural comparison shows that positions related to thermal stability are concentrated at the loop segments of the β-glucosidases (8 out 9 for Sfβgly), whereas positions not related to thermal stability are mainly located in their α-helices and β-strands (5 out 8 for Sfβgly). Naturally, this distribution mainly results from the properties of the proline residues, which favor loop segments [33]. Indeed, these data are in agreement with previous observations demonstrating that proline and charged residues and loop length and mobility are related to the thermal stability of proteins [34], [35]. Thus, based on their similarities, coupled positions related to the thermal stability of β-glucosidases were labeled sector S (Figure 2).

Therefore, in contrast to sectors observed in the serine protease [13] and sector A of β-glucosidases (discussed above), which are composed of residues contacting each other, sector S of the β-glucosidases is formed by positions with no physical interactions but is characterized by the prevalence of proline residues. Because of their placement in loops, sector S positions are jointly related to the thermal stability of β-glucosidases.

## Supporting Information

Figure S1 **SDS-PAGE of the purified wild-type and mutant Sfβgly proteins.** Mutant enzymes are identified by the residue and number of the covariant position followed by A, representing the replacement that they contain. Wt stands for wild type Sfβgly. Details regarding the protein purification procedures are found in the Materials and Methods section. The gels (12% polyacrilamide) were stained using Coomassie blue R-250. Approximately 2 µg of protein was loaded in each lane. MW, molecular weight markers.
(TIF)

Figure S2 **The effect of substrate concentration on the activity of the wild-type and mutant Sfβgly proteins.** Mutant enzymes are identified by the residue and number of the covariant position in which A was introduced. The substrates are *p*-nitrophenyl β-glucoside (NPβglc), *p*-nitrophenyl β-galactoside (NPβgal) and *p*-nitrophenyl β-fucoside (NPβfuc). Substrates were prepared in prepared in 100 mM sodium citrate – sodium phosphate buffer pH 6.0. Experiments were performed at 30°C. Dots are the experimental data. The continuous line represents the values calculated based on the best fitting of the experimental data into the Michaelis-Menten equation. Fitting was performed using EnzFitter software.
(TIF)

Figure S3 **Differential scanning fluorimetry of the wild-type and mutant Sfβgly proteins.** Samples (10 ng) were incubated with SYPRO stain, and the fluorescence was recorded at different temperatures. Mutant enzymes are identified by the residue and number of the covariant position in which A was introduced. Dots are the experimental data, whereas lines represent the calculated values produced based on the best fitting. Fitting process was performed using the software Enzfitter and the equation deduced on [22].
(TIF)

Figure S4 **Structural comparison between the active site of β-glucosidases (A) and 6-phospho β-glucosidases (B) from family GH1.** A glucose unit (gray and red; A) is represented in the glycone subsite of the β-glucosidases Sfβgly (green; Phyre2 model), *Neotermes koshunensis* (blue; PDB 3VIF) and wheat *Triticum aestivum* (yellow; PDB 3AIQ), whereas a 6-phospho glucose unit (gray and red, B) is presented for the 6-phospho β-glucosidases from *Streptococcus pneumonia* (cyan, PDB 4IPN) and *Lactococcus lactis* (formely know as *Streptococcus lactis*) (majenta, PDB 4PBG). Residues numbering was based on Sfβgly. Dotted lines represent hydrogen bonds. 6′-P-OH indicates the phosphate group bound to the glucose hydroxyl 6. Based on [31] and [32].
(TIF)

Table S1 **Mutagenic primer sequences and annealing temperatures.**
(DOCX)

Table S2 **Extinction coefficients and percentage of F, Y and W for wild-type and mutant Sfβgly.**
(DOCX)
