# Supplementary figures and images for: Sets of Covariant Residues Modulate the Activity and Thermal Stability of GH1 β-Glucosidases
Source: PLoS One. 2014 May 7;9(5):e96627. doi: 10.1371/journal.pone.0096627 (PMC4013033; doi:10.1371/journal.pone.0096627)

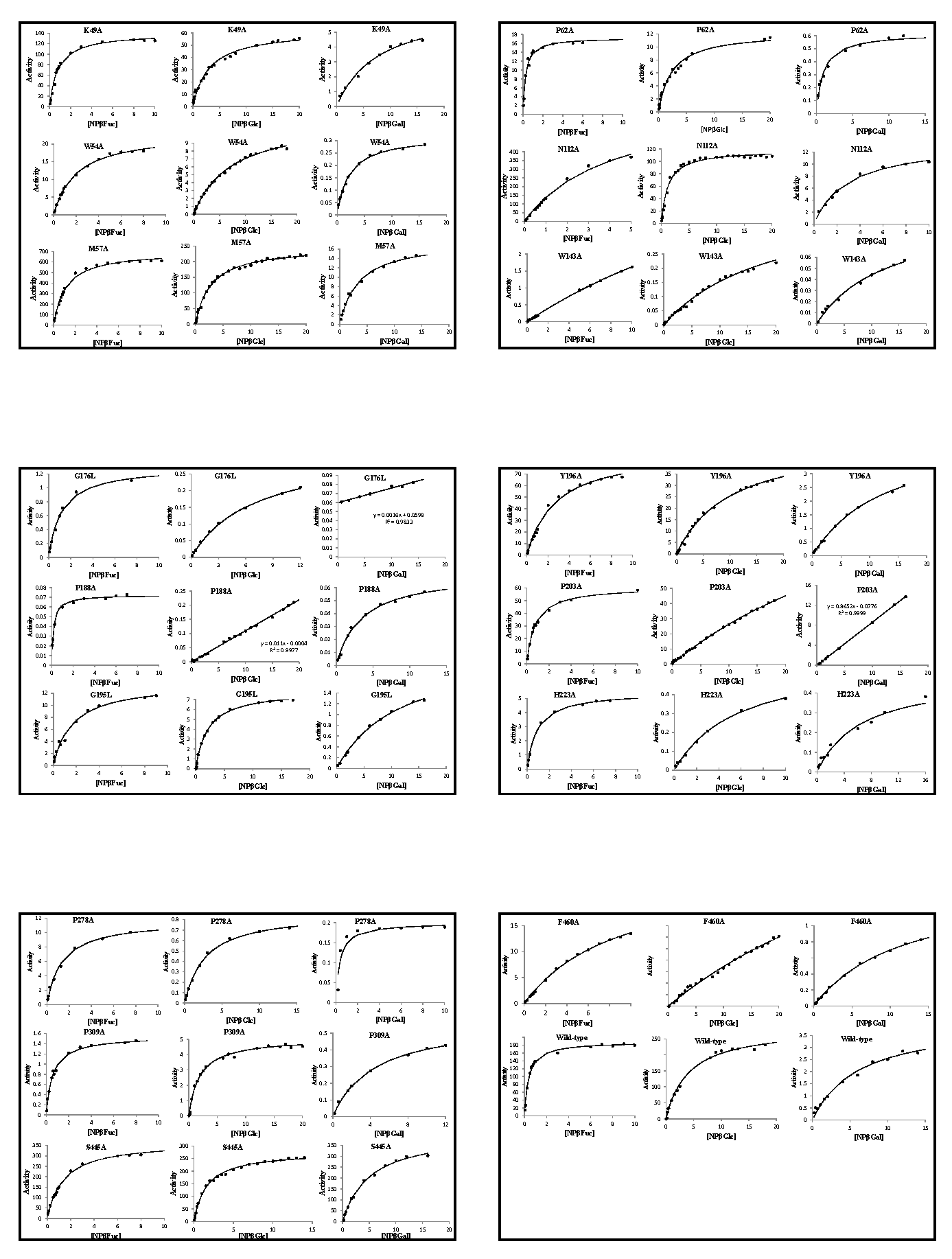

Supplement: Figure S2 — The effect of substrate concentration on the activity of the wild-type and mutant Sfβgly proteins. Mutant enzymes are identified by the residue and number of the covariant position in which A was introduced. The substrates are p-nitrophenyl β-glucoside (NPβglc), p-nitrophenyl β-galactoside (NPβgal) and p-nitrophenyl β-fucoside (NPβfuc). Substrates were prepared in prepared in 100 mM sodium citrate – sodium phosphate buffer pH 6.0. Experiments were performed at 30°C. Dots are the experimental data. The continuous line represents the values calculated based on the best fitting of the experimental data into the Michaelis-Menten equation. Fitting was performed using EnzFitter software. (TIF) [file pone.0096627.s002.tif]

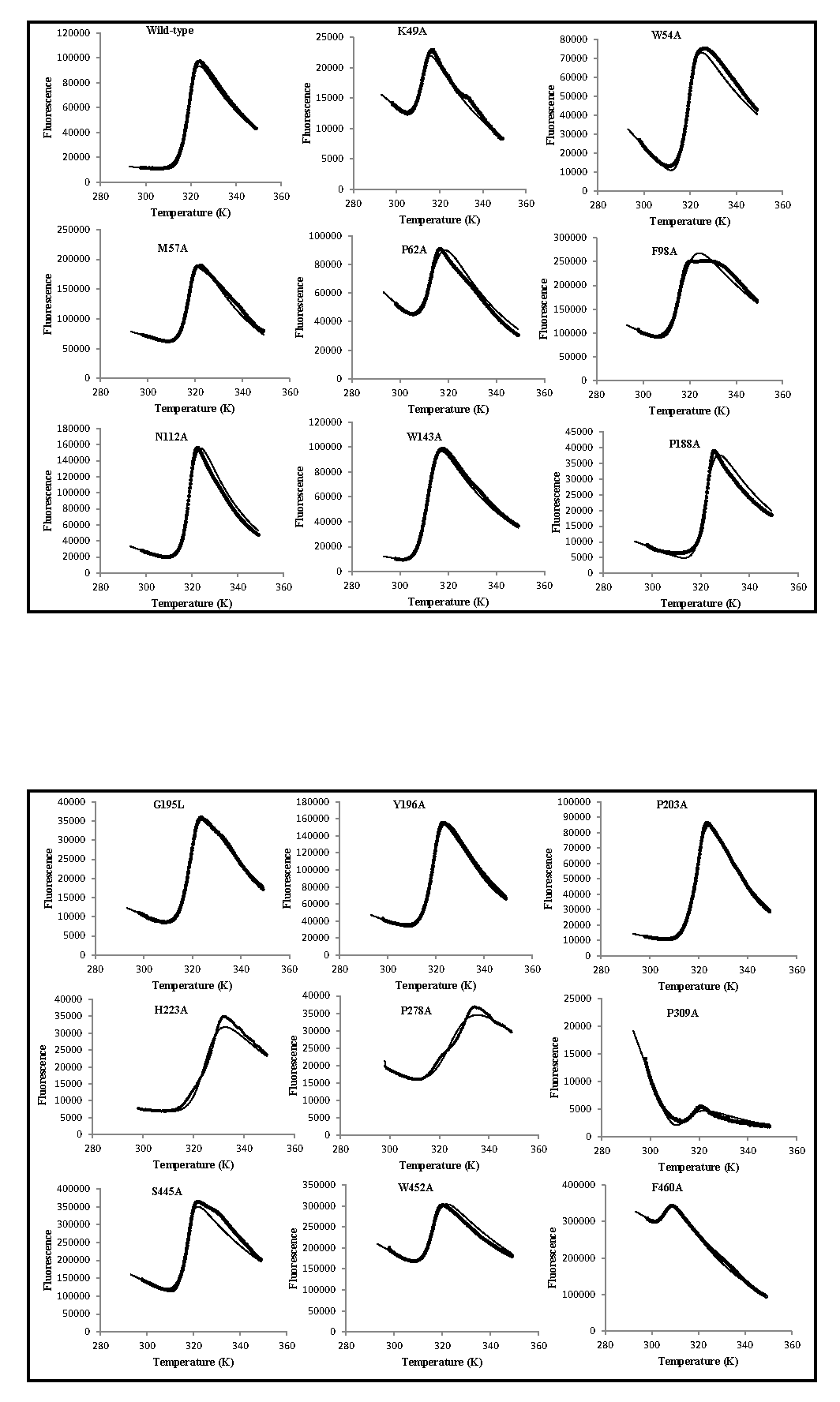

Supplement: Figure S3 — Differential scanning fluorimetry of the wild-type and mutant Sfβgly proteins. Samples (10 ng) were incubated with SYPRO stain, and the fluorescence was recorded at different temperatures. Mutant enzymes are identified by the residue and number of the covariant position in which A was introduced. Dots are the experimental data, whereas lines represent the calculated values produced based on the best fitting. Fitting process was performed using the software Enzfitter and the equation deduced on [22]. (TIF) [file pone.0096627.s003.tif]

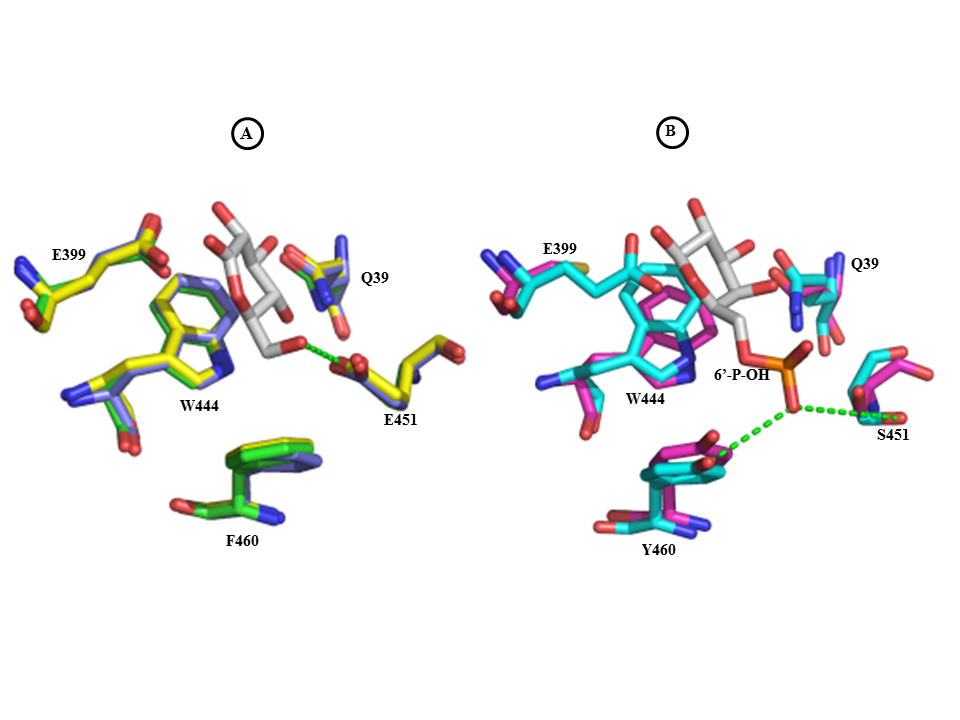

Supplement: Figure S4 — Structural comparison between the active site of β-glucosidases (A) and 6-phospho β-glucosidases (B) from family GH1. A glucose unit (gray and red; A) is represented in the glycone subsite of the β-glucosidases Sfβgly (green; Phyre2 model), Neotermes koshunensis (blue; PDB 3VIF) and wheat Triticum aestivum (yellow; PDB 3AIQ), whereas a 6-phospho glucose unit (gray and red, B) is presented for the 6-phospho β-glucosidases from Streptococcus pneumonia (cyan, PDB 4IPN) and Lactococcus lactis (formely know as Streptococcus lactis) (majenta, PDB 4PBG). Residues numbering was based on Sfβgly. Dotted lines represent hydrogen bonds. 6′-P-OH indicates the phosphate group bound to the glucose hydroxyl 6. Based on [31] and [32]. (TIF) [file pone.0096627.s004.tif]
