# Supplementary material for: Sets of Covariant Residues Modulate the Activity and Thermal Stability of GH1 β-Glucosidases
Source: PLoS One. 2014 May 7;9(5):e96627. doi: 10.1371/journal.pone.0096627 (PMC4013033; doi:10.1371/journal.pone.0096627)
Supplement: Table S1 — Mutagenic primer sequences and annealing temperatures. (DOCX) [file pone.0096627.s005.docx]

**Supplementary table S1** – Mutagenic primer sequences and annealing temperatures

| Position | Direction | Primer sequence | Tm (°C) |
| --- | --- | --- | --- |
| 49 | FW | 5’ GAA GAT GGT GCG GGT GAA AAC 3’ | 56.8 |
| 49 | RV | 5’ GTT TTC AAC CGC ACC ATC TTC 3’ | 54.8 |
| 54 | FW | 5’ GAA AAC ATC GCG GAC TAC ATG 3’ | 54.1 |
| 54 | RV | 5’ CAT GTA GTC CGC GAT GTT TTC 3’ | 54.1 |
| 57 | FW | 5’ TGG GAC TAC GCG GTC CAT AAC 3’ | 58.9 |
| 57 | RV | 5’ GTT ATG GAC CGC GTA GTC CCA 3’ | 58.9 |
| 62 | FW | 5’ CAT AAC ACC GCA GAA GTC ATC 3’ | 53.6 |
| 62 | RV | 5’ GAT GAC TTC TGC GGT GTT ATG 3’ | 53.6 |
| 98 | FW | 5’ GCT TAC AGG GCC TCC CTC TCC 3’ | 61.7 |
| 98 | RV | 5’ GGA GAG GGA GCG CCT GTA AGC 3’ | 62.2 |
| 112 | FW | 5’ GGC ATG GCC GCT GAG GTC AAC 3’ | 63.7 |
| 112 | RV | 5’ GTT GAC CTC AGC GGC CAT GCC 3’ | 63.7 |
| 143 | FW | 5’ CTC TAC CAT GCG GAT CTT CCT 3’ | 56.1 |
| 143 | RV | 5’ AGG AAG ATC CGC ATG GTA GAG 3’ | 56.1 |
| 176 | FW | 5’ GAG AAC TTC CTA GAC AGA GTC 3’ | 51.5 |
| 176 | RV | 5’ GAC TCT GTC TAG GAA GTT CTC 3’ | 51.5 |
| 188 | FW | 5’ TTC AAC GAG GCT AGA GAG ATT 3’ | 53.0 |
| 188 | RV | 5’ AAT CTC TCT AGC CTC GTT GAA 3’ | 53.0 |
| 195 | FW | 5’ TGC TTT GAG CTT TAT GGA TCT 3’ | 51.9 |
| 195 | RV | 5’ AGA TCC ATA AAG CTC AAA GCA 3’ | 51.9 |
| 196 | FW | 5’ TTT GAG GGT GCT GGA TCT GCT 3’ | 58.9 |
| 196 | RV | 5’ AGC AGA TCC AGC ACC CTC AAA 3’ | 58.9 |
| 203 | FW | 5’ ACC AAG GCT GCG ATC CTA AAC 3’ | 57.6 |
| 203 | RV | 5’ GTT TAG GAT CGC AGC CTT GGT 3’ | 57.6 |
| 223 | FW | 5’ GTG ACT GCT GCT GCC AAG GCT 3’ | 62.9 |
| 223 | RV | 5’ AGC CTT GGC AGC AGC AGT CAC 3’ | 62.9 |
| 278 | FW | 5’ TAC GCT CAT GCT ATC TTC TCA 3’ | 53.1 |
| 278 | RV | 5’ TGA GAA GAT AGC ATG AGC GTA 3’ | 53.1 |
| 309 | FW | 5’ TCT CGT CTG GCA GAA TTC ACT 3’ | 55.9 |
| 309 | RV | 5’ AGT GAA TTC TGC CAG ACG AGA 3’ | 55.9 |
| 329 | FW | 5’ ATC GGA GTG GCC CAC TAC ACA 3’ | 60.5 |
| 329 | RV | 5’ TGT GTA GTG GGC CAC TCC GAT 3’ | 60.5 |
| 398 | FW | 5’ TTC TAC ATC GCT GAG AAT GGC 3’ | 54.8 |
| 398 | RV | 5’ GCC ATT CTC AGC GAT GTA GAA 3’ | 54.8 |
| 445 | FW | 5’ ATG GCT TGG GCT CTA ATG GAC 3’ | 57.3 |
| 445 | RV | 5’ GTC CAT TAG AGC CCA AGC CAT 3’ | 57.3 |
| 449 | FW | 5’ CTA ATG GAC GCC TTT GAA TGG 3’ | 54.0 |
| 449 | RV | 5’ CCA TTC AAA GGC GTC CAT TAG 3’ | 54.0 |
| 451 | FW | 5’ GAC AAC TTT GCA TGG ATG GAG 3’ | 54.2 |
| 451 | RV | 5’ CTC CAT CCA TGC AAA GTT GTC 3’ | 54.2 |
| 452 | FW | 5’ AAC TTT GAA GCG ATG GAG GGT 3’ | 56.8 |
| 452 | RV | 5’ ACC CTC CAT CGC TTC AAA GTT 3’ | 56.8 |
| 456 | FW | 5’ ATG GAG GGT GCT ATT GAG CGT 3’ | 58.6 |
| 456 | RV | 5’ ACG CTC AAT AGC ACC CTC CAT 3’ | 58.6 |
| 460 | FW | 5’ ATT GAG CGT GCC GGC CTT TAC 3’ | 60.7 |
| 460 | RV | 5’ GTA AAG GCC GGC ACG CTC AAT 3’ | 60.7 |
