# Supplementary material for: Sets of Covariant Residues Modulate the Activity and Thermal Stability of GH1 β-Glucosidases
Source: PLoS One. 2014 May 7;9(5):e96627. doi: 10.1371/journal.pone.0096627 (PMC4013033; doi:10.1371/journal.pone.0096627)
Supplement: Table S2 — Extinction coefficients and percentage of F, Y and W for wild-type and mutant Sfβgly. (DOCX) [file pone.0096627.s006.docx]

**Supplementary table S2 –** Extinction coefficients and percentage of F, Y and W for wild-type and mutant Sfβgly

| **Enzyme** | **Extinction coefficient** | **% (W + Y + F)** |
| --- | --- | --- |
| Wild-type | 2,146 | 14,1 |
| K49A | 2,149 | 14,1 |
| W54A | 2,053 | 13,9 |
| M57A | 2,149 | 14,1 |
| P62A | 2,147 | 14,1 |
| N112A | 2,148 | 14,1 |
| W143A | 2,053 | 13,9 |
| P188A | 2,147 | 14,1 |
| G195L | 2,144 | 14,1 |
| Y196A | 2,123 | 13,9 |
| P203A | 2,147 | 14,1 |
| H223A | 2,149 | 14,1 |
| P278A | 2,147 | 14,1 |
| P309A | 2,147 | 14,1 |
| S445A | 2,147 | 14,1 |
| F460A | 2,149 | 13,9 |
